# Comment on Fernández-Vigo et al. Objective Classification of Glistening in Implanted Intraocular Lenses Using Optical Coherence Tomography: Proposal for a New Classification and Grading System. *J. Clin. Med.* 2023, *12*, 2351

**DOI:** 10.3390/jcm12113685

**Published:** 2023-05-26

**Authors:** Nick Stanojcic, Chris Hull, David O’Brart

**Affiliations:** 1 Department of Ophthalmology, St Thomas’ Hospital, London SE1 7EH, UK; 2 Cataract and Cornea Research Group, King’s College London, Strand, London WC2R 2LS, UK; 3 Centre for Applied Vision Research, School of Health Sciences, University of London, London EC1V 0HB, UK

We read with interest your article describing a new objective method for evaluating glistenings in intraocular lenses (IOLs) in vivo [1].

We note that the number of microvacuoles (MV) detected by this method in your study was significantly less than that which we found with the same AcrySof lens material [2]. In your lowest severity group ‘0’ (63.3% of the eyes), you report fewer than 5 MV per entire lens optic section (an approximate 3 mm^2^ area as you have noted). Our lowest grade similarly comprised 59% of eyes, but our MV density was 1–10 MV per mm^2^. Similarly, your highest severity group 4 (~10% eyes) was reported as having >30 MV per IOL section, equivalent to 10 MV per mm^2^. Our groups 4 and above combined, similarly accounted for ~10% of eyes. However, our MV density for groups 4 and above was >31 MVs per mm^2^.

Furthermore, a laboratory study conducted by our group found that the same IOL as in your study (AcrySof SN60WF, Alcon Inc., Fort Worth, TX, USA) demonstrated that the largest proportion of glistenings was between 1–20 micron in diameter (median size 23.8 microns; average density 71 MV/mm^2^) [3]. Another study also found that AcrySof IOL glistenings were relatively small compared to other IOLs (6.2 microns) [4].

We hence suspect that a large number of MVs may have been undetected by the OCT method, in particular the smallest ones. This may be due to the nature of the swept source OCT, the resolution of which you quoted as 8 microns axial and 20 microns transverse. It is therefore possible that the transverse resolution may not be sufficient to detect smaller glistenings whose diameters are less than 20 microns. Despite the significant advances in swept source OCT technology compared to standard-domain OCT, there is an inherent variability of scanning due to eye and/or patient motion and the reliance on software to interpolate data between the scans [5].

The measurement of glistenings with slit-lamp methods relates to light traversing the pupil and can therefore be related to an effect on vision. The slit-lamp methods cause reflections from glistenings within the illuminated volume of the IOL (rather than a section) and the detection of glistenings is dependent on their luminance contrast and not size.

OCT methods will only detect hyper-reflective foci within a very thin section. This is because the axial resolution of 8 microns produces a very shallow effective depth of focus, extending minimally on either side of the plane of the section. If, however, there was a proven correlation between the glistenings density from OCT sections and the entire volume of the IOL, then the OCT section may act as a surrogate measure; however, this has not been tested.

We also note the lack of detail regarding the method used for what appears to be a subjective counting of glistenings in your study. In particular, we would be interested to know whether the display screen luminance and ambient lighting were standardized when glistenings were counted by your team. In our experience, this can have an effect on the results. Additionally, as with retinal OCT imaging, it may be that artefacts on the IOL surface, such as the one resulting in a high-intensity signal in your figure ‘1B’, may impair the detection of underlying MVs.

We propose that the underestimation of the number of MVs by SS-OCT may be due to a limitation of this technology or the subjective method used for counting MVs. We recommend that the authors of the article consider validating their OCT method with a standard digital slit-lamp photography method.
